# Do Sexually Satisfied Individuals Think That They Live Longer? Results from the German Ageing Survey

**DOI:** 10.3390/healthcare10122482

**Published:** 2022-12-08

**Authors:** André Hajek, Elzbieta Buczak-Stec, Hans-Helmut König

**Affiliations:** Department of Health Economics and Health Services Research, University Medical-Center Hamburg-Eppendorf, Hamburg Center for Health Economics, 20246 Hamburg, Germany

**Keywords:** longevity, perceived longevity, sexual satisfaction, subjective life expectancy, mortality, Germany, satisfaction with sex life, older adults, gender differences, satisfaction, sexual health

## Abstract

The aim of this study was to determine the association between sexual satisfaction and expected longevity among middle-aged and older adults (also stratified by sex). Data were taken from the German Ageing Survey (year 2011; n = 3231)—a nationally representative sample of community-dwelling individuals ≥ 40 years in Germany. A widely used question was used to quantify sexual satisfaction. Furthermore, the expected life expectancy served as an outcome measure. After adjusting for various covariates, multiple linear regressions showed that sexual satisfaction was associated with higher expected longevity among the total sample (β = 0.28, *p* < 0.05). Moreover, it was associated with higher expected longevity among women (β = 0.48, *p* < 0.05), but not men. In conclusion, adjusting for several covariates, our results showed that there is an association between sexual satisfaction and higher expected longevity, particularly in women. Efforts to increase sexual satisfaction may thus also contribute to expected longevity which, in turn, can be beneficial for actual longevity.

## 1. Introduction

Sexuality in late life has long been disregarded by scientific disciplines. However, there is growing attention in this research area [1]. Despite the prevalence of sexual disorders [2], many older individuals engage in sexual activity [3]. Furthermore, a high proportion is satisfied with their sex life [1]. For example, in Germany in the year 2002, the average sexual satisfaction (5-point scale from 1 (worst) to 5 (best)) was 3.8 (SD: 1.0) among community-dwelling individuals aged 40 to 64 years and it was 3.3 (SD: 0.9) among community-dwelling individuals aged 65 years and over [1].

Such sexual satisfaction is not limited to sexual intercourse, but can also include, e.g., kissing and caressing. An earlier study actually showed that participants in a Polish study aged 60 to 81 were most likely to practice more subtle forms of sexual activity instead of intercourse [4].

Sexual satisfaction is a key component of one’s own later life. It is closely connected to sexual health [5]. Furthermore, and more broadly, higher sexual satisfaction can contribute to increased subjective well-being including higher positive affect, lower negative affect and higher satisfaction with life among younger [6], as well as among middle-aged and older adults [7] in Germany over time. Overall, subjective well-being can contribute to successful ageing [8].

While there is increasing knowledge regarding consequences of sexual satisfaction for many parts of an individual’s life, it remains completely unknown how sexual satisfaction is associated with expected longevity. Longevity expectations (often synonymously: subjective life expectancy or perceived nearness/distance to death) refer to one’s own perception of longevity. For example, a former study showed that individuals aged 40 and over in Germany had a perceived life expectancy of nearly 85 years [9].

We assume that individuals scoring high in sexual satisfaction think that they live longer compared to individuals scoring low in sexual satisfaction. In an explorative fashion, we assume that a higher sexual satisfaction could reflect underlying factors such as enjoyment of life, living an active life, a high quality of relationship in partnership, a potentially good health status (both, oneself and of the partner) or the feeling of being young. Individuals who are satisfied with their sex life may also think that they are generally better off than individuals who have a low satisfaction with their sex lives. These positive comparisons (in terms of sexual satisfaction) may also contribute to a higher expected longevity. Moreover, an individual with a high sexual satisfaction may also think that his/her partner has a good future health status. Thus, his/her partner would take care of the individual in the future if he or she needed care, which, in turn, can contribute to a higher expected longevity.

We think that understanding the association between sexual satisfaction and expected longevity is relevant due to the fact that a low expected longevity can reflect a self-fulfilling prophecy [9] and can reduce future health (via adverse lifestyle [10]). For instance, prior research showed an association between expected death and distress [11]. Hence, following former research [9], we assume that expected longevity can influence future chronic conditions, stressing the importance of our topic. Moreover, it should be emphasized that sexual satisfaction (our independent variable of interest) is modifiable in middle and even old age [1].

Due to the missing previous studies examining this association of interest, our study is explorative in its manner. The aim of this study was as follows: To investigate the association between sexual satisfaction and expected longevity. Furthermore, we were interested in whether such an association differs between men and women.

## 2. Materials and Methods

### 2.1. Sample

Secondary data was used for our current study. More precisely, this study employed cross-sectional data (wave 4, year 2011) from the German Ageing Survey (DEAS). The DEAS study is a nationally representative sample of individuals in private households in Germany aged 40 and up (second half of life). It is funded by the Federal Ministry for Family Affairs, Senior Citizens, Women and Youth (BMFSFJ).

Individuals were drawn using a national probability sampling method. Generally, the DEAS study’s key inclusion condition was that participants must be at least 40 years old and live in a private household. Individuals living in institutionalized situations were therefore excluded. Starting in the year 1996, this study has a cohort-sequential design. Topics cover, for example, subjective well-being, retirement transitions, ageism, or media consumption.

First, individuals were interviewed at their homes (regarding general topics such as sociodemographic questions). Subsequently, individuals could fill out a drop-off questionnaire including more sensitive questions (such as well-being or loneliness). The drop-off questionnaire was completed by 4005 individuals in the fourth wave. In this wave, the response rate was 56%. Health difficulties and refusal were the most common causes for the lack of follow-up data. About 8% of these 4005 individuals (n = 338) did not answer to our outcome measure. Furthermore, during data cleaning operations, around 0.3% of the values (n = 13) were eliminated. Another two cases were eliminated due to a lack of credibility (see the next section). For reasons of missing values in the independent variables, the total analytical sample for our current study was 3231 observations. Klaus et al. [12] provided more information about the DEAS study. It is worth noting that wave 4 of the DEAS study was used for reasons of data availability. More precisely, this was the last wave in which sexual satisfaction was included.

With regard to sample selection bias: In sum, 4854 individuals were interviewed in wave 4 and 3231 individuals were included in the analytical sample. While the average age was 66.6 years (SD: 11.8 years) among the individuals not included in the analytical sample, it was 64.6 years (SD: 10.6 years) in the analytical sample (*p* < 0.001; Cohen’s d = 0.18). The proportion of women was 52.0% among the individuals not included in the analytical sample, whereas it was 48.6% in the analytical sample (*p* = 0.02, Cramer’s V = 0.03). Moreover, the proportion of individuals with a high education was 36.8% among the individuals not included in the analytical sample, whereas it was 42.2% in the analytical sample (*p* < 0.001, Cramer’s V = 0.09). Moreover, while the average self-rated health was 2.6 (SD: 0.9) among the individuals not included in the analytical sample, it was 2.5 (SD: 0.8) in the analytical sample (*p* < 0.001; Cohen’s d = 0.19). The average household net equivalence income in Euro was 1745.8 (SD: 1626.0) among the individuals not included in the analytical sample, whereas it was 1872.9 (SD: 1381.2) in the analytical sample (*p* < 0.01; Cohen’s d = −0.09).

Written informed consent was given by all participants prior to the interview. The DEAS study is in compliance with the Helsinki Declaration. It should be mentioned that an ethics statement for this study was not necessary because the criteria for the need of an ethical statement were not met (e.g., lack of information about the aims of the study, risk for the respondents, examination of patients).

### 2.2. Dependent Variable

Similar to other large cohort studies, individuals were asked to assess their expected longevity. More precisely, the wording was as follows: ‘What age do you think you will live to?’ [_ _ _ years]. Implausible values were removed. More exactly, two individuals were removed because they reported an expected longevity of 200 years and above (200 years and 234 years). Additionally, it is worth noting that no individual was removed because his or her expected longevity was below their current chronological age. Furthermore, four individuals reported an expected longevity below 200 years, but above 120 years (136 years, 150 years, 170 years, 180 years). Considering that the highest verified ages are about 120 years, it can be critically evaluated whether these are rational expectations. Thus, we dropped these four individuals in a robustness check.

### 2.3. Independent Variables

In line with former research [13,14], a single item measure was used to measure sexual satisfaction. Thus, the respondents were asked to report their satisfaction with their sex life (from 1 = very dissatisfied to 5 = very satisfied).

In accordance with prior research and using theoretical considerations [15,16], it was adjusted for several covariates in regression analysis: Age, sex, marital status (never married; divorced; widowed; married, living separated from spouse; married, living together with spouse), labor force participation (not employed; retired; employed), educational level (ISCED-classification [17]): low (0–2), medium (3–4), and high (5–6) education), and (log) household net equivalence income (in Euro).

Moreover, health-related covariates were included in regression analysis as follows: self-rated health (single item, from 1 = very good to 5 = very bad), depressive symptoms (15-item version of the Centre for Epidemiologic Studies [18], sum score ranging from 0 to 45, with higher values reflecting more depressive symptoms), physical functioning (SF-36 subscale physical functioning [19], ranging from 0 (worst) to 100 (best)), and a count score for physical conditions (0 to 11 chronic conditions: cardiac and circulatory disorders; bad circulation; joint, bone, spinal and back problems; respiratory problems, asthma, shortness of breath; stomach and intestinal problems; cancer; diabetes; gall bladder, liver or kidney problems; bladder problems; eye problems, vision impairment; ear problems, hearing problems).

### 2.4. Statistical Analysis

Sample characteristics for our analytical sample (also stratified by sex) were first calculated. In the next step, multiple linear regressions were conducted (also stratified by sex) to examine the association between sexual satisfaction and expected longevity. Additionally, it may be worth noting that sex-stratified regressions were conducted in an explorative manner.

To handle missing values, we used full-information maximum likelihood (FIML) in a robustness check [20]. The level of statistical significance was set at *p* < 0.05. Stata 16.1 (StataCorp., College Station, TX, USA) was used to conduct the analyses.

## 3. Results

### 3.1. Sample Characteristics

Sample characteristics for the analytical sample (also stratified by sex) are given in Table 1. In our analytical sample, the average age equaled 64.6 years (SD: 10.6 years), ranging from 43 to 95 years. Moreover, 48.6% of the individuals were female. The expected longevity was 83.6 years (SD: 7.4 years) in the total sample (men: 84.1 years, SD: 7.5 years; women: 83.1 years, SD: 7.5 years). Additionally, average sexual satisfaction was 3.4 (SD: 0.9) in the total sample (among men: 3.3, SD: 1.0; among women: 3.4, SD: 0.9). Additional details are given in Table 1.

### 3.2. Regression Analysis

Findings of multiple linear regressions are shown in Table 2. R² values ranged from 0.11 (among men) to 0.13 (among women). Average variance inflation factor (VIF) was 1.87 and all VIFs were below 5, indicating the absence of multicollinearity. It was adjusted for age, family status, labor force participation, education, income, body-mass-index, physical functioning, self-rated health, and chronic diseases in multiple linear regressions (with robust standard errors).

After adjusting for various covariates, multiple linear regressions showed that sexual satisfaction was associated with higher expected longevity among the total sample (β = 0.28, *p* < 0.05). Moreover, it was associated with higher expected longevity among women (β = 0.48, *p* < 0.05), but not men (β = 0.13, *p* = 0.50).

We also did a robustness check where FIML was used to address missing data. Our findings remained nearly the same. More precisely, multiple linear regressions showed that sexual satisfaction was associated with higher expected longevity among the total sample (β = 0.27, *p* < 0.05), women (β = 0.48, *p* < 0.05), but not men (β = 0.12, *p* = 0.51).

Moreover, in another robustness check we restricted the expected longevity to a maximum expected longevity of 120 years. However, findings remained nearly identical: multiple linear regressions showed that sexual satisfaction was associated with higher expected longevity among the total sample (β = 0.29, *p* < 0.05), women (β = 0.51, *p* < 0.05), but not men (β = 0.14, *p* = 0.46).

## 4. Discussion

Based on data from a large, nationally-representative sample, our aim was to determine the association between sexual satisfaction and expected longevity among middle-aged and older adults. Regressions showed that sexual satisfaction was associated with higher expected longevity among the total sample, and women, but not men. These associations remained nearly the same in robustness checks. To the best of our knowledge, this is the very first study investigating the association between sexual satisfaction and expected longevity. Therefore, it is difficult to compare our findings to former studies.

As outlined in the introduction, we assume that a higher sexual satisfaction may reflect factors such as, enjoying life, sense of youthfulness, and a generally active lifestyle. Moreover, a high sexual satisfaction may reflect a good partnership. Additionally, it may indicate a high self-rated health of one’s own, which may contribute to a higher expected longevity. In particular, the good health of a partner may lead to the increased expected longevity of one’s self. Thus, the respondent may think that their partner can take care of her or him in the future. This may also contribute to a higher expected longevity. Moreover, positive sexual comparisons (i.e., an individual with a high sexual satisfaction may think that are better off—also in terms of overall health—than other individuals in their age group) may contribute to a higher expected longevity.

It should be noted that while sexual satisfaction was positively associated with expected longevity among women, it was not associated with expected longevity among men in our study. A possible, but speculative explanation for the association among middle-aged and older women may be that sexual satisfaction is a particularly important part of female lives, making them “feel alive”. Such feelings may drive expected longevity. Another possible explanation may be that middle-aged men, in particular, think that work-related factors (such as job-related stress or working hours per week) may contribute to their expected longevity. Moreover, it may be the case that men are less willing to report poor sexual performance in later life and consequently may deny its importance for perceived longevity. However, these are quite speculative explanations which certainly require future research (e.g., based on qualitative designs such as in-depth interviews).

Some strengths and limitations are worth noting. This is the first study focusing on the association between sexual satisfaction and expected life expectancy. Data were taken from the nationally representative DEAS study covering community-dwelling individuals aged 40 years and over. Moreover, it was adjusted for several covariates. We also performed sensitivity analysis (e.g., using FIML). However, some limitations are also worth acknowledging. The current study is a cross-sectional one with its known shortcomings in terms of directionality. For example, expected life expectancy could contribute to sexual satisfaction. Moreover, as is common in large cohort studies, single-item measures were used to quantify sexual satisfaction and expected life expectancy. For example, by using a tool for self-rated sexual satisfaction, we cannot dismiss the possibility that some respondents are satisfied even though they have no sexual activity at all. Thus, future research with more sophisticated tools would be desirable to confirm our current results. Additionally, a rather small sample selection bias has been identified in the DEAS study [12]. Given the large sample size, there were actually some significant differences in socioeconomic and health-related factors between individuals included in our analytical sample and the remaining individuals (see the sample section for further details). In terms of effect size, however, these differences were mostly negligible or small.

## 5. Conclusions and Future Research

In conclusion, after adjusting for several covariates, our results show that there is an association between sexual satisfaction and higher expected longevity, particularly in women. Efforts to increase sexual satisfaction may thus also contribute to expected longevity, which, in turn, can be beneficial for actual longevity.

With regard to future research, upcoming studies are required to clarify the underlying mechanisms. Moreover, future studies with a longitudinal design are needed to clarify the directionality. Qualitative studies in this area are also recommended. Additionally, moderating factors (e.g., educational level or personality traits) in this relationship should be explored.

## Figures and Tables

**Table 1:** Sample characteristics.

Variables	Total Sample	Men	Women
	N = 3231	N = 1662	N = 1569
Expected longevity (in years): Mean (SD); Range	83.6 (7.4); 48–180	84.1 (7.5); 60–180	83.1 (7.4); 48–170
Sexual satisfaction (ranging from 1 = very unsatisfied to 5 = very satisfied): Mean (SD)	3.4 (0.9)	3.3 (1.0)	3.4 (0.9)
Age: Mean (SD)	64.6 (10.6)	65.8 (10.7)	63.3 (10.3)
Marital status: N (%)			
Married, living together with spouse	2391 (74.0%)	1342 (80.7%)	1049 (66.9%)
Married, living separated from spouse	42 (1.3%)	21 (1.3%)	21 (1.3%)
Divorced	288 (8.9%)	113 (6.8%)	175 (11.2%)
Widowed	343 (10.6%)	102 (6.1%)	241 (15.4%)
Single	167 (5.2%)	84 (5.1%)	83 (5.3%)
Education (ISCED-97): N (%)			
Low education	212 (6.6%)	45 (2.7%)	167 (10.6%)
Medium education	1656 (51.3%)	789 (47.5%)	867 (55.3%)
High education	1363 (42.2%)	828 (49.8%)	535 (34.1%)
Employment status: N (%)			
Working	1114 (34.5%)	557 (33.5%)	557 (35.5%)
Retired	1799 (55.7%)	993 (59.7%)	806 (51.4%)
Other (not employed)	318 (9.8%)	112 (6.7%)	206 (13.1%)
Household net equivalence income (in Euro): Mean (SD)	1872.9 (1381.2)	1961.7 (1530.7)	1778.9 (1196.2)
Body-Mass-Index: Mean (SD)	27.0 (4.4)	27.1 (3.8)	26.8 (5.0)
Physical functioning (from 0 (worst) to 100 (best)): Mean (SD)	83.5 (21.8)	85.1 (21.0)	81.7 (22.5)
Self-rated health (from 1 = very good to 5 = very bad): Mean (SD)	2.5 (0.8)	2.5 (0.8)	2.4 (0.8)
Number of physical illnesses (count: 0 to 11 physical illnesses): Mean (SD)	2.5 (1.8)	2.5 (1.9)	2.4 (1.8)

**Table 2:** Sexual satisfaction and expected longevity. Results of multiple linear regressions (wave 4).

	Expectations of Longevity—Total Sample	Expectations of Longevity—Men	Expectations of Longevity—Women
Covariates ^†^	✓	✓	✓
Sexual satisfaction (ranging from 1 = very unsatisfied to 5 = very satisfied)	0.28 * (0.004–0.55)	0.13 (−0.24–0.49)	0.48 * (0.07–0.90)
Observations	3231	1662	1569
(Pseudo) R²	0.12	0.11	0.13

Comments: Beta coefficients are displayed. 95% CI in parentheses. * *p* < 0.05. ^†^ Covariates cover (✓): age, family status, labor force participation, education, income, body-mass-index, physical functioning, self-rated health, and chronic diseases.

## Data Availability

The data used in this study are third-party data. The anonymized data sets of the DEAS (1996, 2002, 2008, 2011, 2014, 2017, 2020, 2020/2021) are available for secondary analysis. The data has been made available to scientists at universities and research institutes exclusively for scientific purposes. The use of data is subject to written data protection agreements. Microdata of the German Ageing Survey (DEAS) are available free of charge to scientific researchers for non-profitable purposes. The FDZ-DZA provides access and support to scholars interested in using DEAS for their research. However, for reasons of data protection, signing a data distribution contract is required before data can be obtained. For further information on the data distribution contract, please see https://www.dza.de/en/research/fdz/access-to-data/formular-deas-en-english (accessed on 10 November 2022).
